# Spatial transcriptomic analysis of kidney biopsies identifies activation of complement and SPP1 networks in *Staphylococcus* infection-associated glomerulonephritis

**DOI:** 10.3389/fneph.2026.1863912

**Published:** 2026-07-17

**Authors:** Spyros Karaiskos, Luis Santana-Quintero, Cherri Bott, Rahul Paul, Sergey V. Brodsky, Isabelle Ayoub, Samir V. Parikh, Brad H. Rovin, Tibor Nadasdy, Hira L. Nakhasi, Sreenivas Gannavaram, Anjali A. Satoskar

**Affiliations:** 1 Division of Analytics & Benefit-Risk Assessment, Office of Biostatistics and Phramacovigilance Center for Biologics Evaluation and Research, Food and Drug Administration, Silver Spring, MD, United States; 2 Department of Pathology, Renal and Transplant Pathology Division, The Ohio State University, Columbus, OH, United States; 3 Department of Internal Medicine, Nephrology Division, The Ohio State University, Columbus, OH, United States; 4 Division of Emerging and Transfusion Transmitted Diseases, Office of Blood Research and Review Center for Biologics Evaluation and Research, Food and Drug Administration, Silver Spring, MD, United States

**Keywords:** Secreted phosphoprotein 1 (SPP1), vascular endothelial growth factor (VEGF), Angiopoietin like proteins (ANG PTL), protease activated receptors (PARs), Placental growth factor (PGF), Integrin alpha X (ITGAX), Parietal epithelial cells (PECs), complement

## Abstract

**Background:**

*Staphylococcus* infection-associated glomerulonephritis (SAGN) has emerged as the leading cause of infection-related glomerulonephritis (IRGN) in the Western subcontinents, but pathogenesis remains poorly understood in the absence of animal models and detailed molecular mechanisms.

**Methods:**

To dissect the complex underlying immunopathology, we analyzed formalin-fixed paraffin-embedded kidney biopsy tissues from three patients with SAGN and one normal control kidney (NCK) using single-cell spatial transcriptomics (analyzing a total of 3,993 cell-specific transcriptomes). The SAGN biopsies showed diffuse endocapillary hypercellularity with focal crescents. Following annotation of various cell types, we constructed cell communication networks using CellChat to identify ligand–receptor interactions in normal and SAGN kidneys.

**Results:**

Secreted phosphoprotein 1 (SPP1/osteopontin) emerged as the most enriched signaling network in SAGN with markedly increased autocrine and paracrine SPP1–CD44 activity among tubular segments and parietal epithelial cells (PECs). In contrast, SPP1-integrin signaling was mainly observed in healthy control kidneys. Consistent with previous reports, complement pathway activation was detected exclusively in diseased kidneys, with PECs as principal signal receivers, suggesting the potential role in crescent formation. Transcripts for C2, C3, CD55, Factor B, and Factor D showed significant upregulation in SAGN. In contrast, vascular endothelial growth factor (VEGF), angiopoietin-like proteins (ANG PTL), and progranulin/granulin networks exhibited decreased communication probabilities in SAGN versus NCK, suggesting the suppression of protective angiogenic and anti-inflammatory signaling. Immunofluorescence staining for SPP1 showed increased staining in renal tubular segments in SAGN biopsies, supporting our transcriptomic data.

**Conclusion:**

SPP1 and complement pathways appear to drive pro-inflammatory responses, while protective VEGF signaling is compromised, providing potential therapeutic targets for this disease.

## Highlights

- This is a pilot study using single-cell spatial transcriptomics with CellChat analysis on SAGN and normal control kidney biopsy samples.
- SPP1 (osteopontin) was the top enriched signaling network along with significantly increased complement network communication in SAGN kidneys.
- VEGF, Granulin/Progranulin, PPARs, and Angiopoietin-like protein networks show decreased communication probabilities in SAGN kidneys.

## Introduction

Infection-related glomerulonephritis (IRGN) is considered a renal-limited autoimmune sequela of recent or ongoing bacterial infection presenting with acute nephritis and renal dysfunction (1–5), but typically no primary infection in the kidney. There is remarkable microbiologic diversity associated with the development of IRGN, ranging from Gram-positive pyogenic cocci, Gram-negative bacilli, to zoonotic pathogens like *Bartonella* and *Coxiella* species (6, 7). Acute *Staphylococcus* infection-associated glomerulonephritis (SAGN) has emerged as the leading cause of IRGN over the past two decades in Western subcontinents (amid other Gram-positive cocci and Gram-negative zoonotic pathogens) (2, 3, 6, 7). Granular IgA and C3-containing glomerular immune complex deposits, endocapillary hypercellularity, and complement consumption are the key pathologic features, often overlapping with other glomerulonephritides (IgAN, C3 GN, and post-streptococcal GN) (3–5, 8–10). Beyond that, not much is known about the immunopathology of SAGN; no animal models and no treatment protocols beyond infection control are available (11, 12). Glomerular lesions may persist despite antibiotic treatment, leading to chronic kidney disease (9).

This is a pilot study to investigate the complex immunopathology underlying SAGN and help in the molecular interrogation of kidney resident cell groups and immune cells. We applied single-cell spatial transcriptomic analysis on formalin-fixed paraffin-embedded (FFPE) sections of SAGN kidney biopsies to identify enriched gene networks and their communication probabilities using ligand–receptor interactions. Our findings may also illuminate overlapping networks in other GNs.

## Materials and methods

### Tissue preparation for the Visium CytAssist instrument

FFPE tissue blocks from four biopsy tissues samples [SAGN, *n* = 3 and baseline zero-time living donor transplant biopsy representing normal control kidney (NCK), *n* = 1] were sectioned at 5-µm thickness. All three samples of SAGN kidneys included in this study were cases with endocapillary hypercellularity with focal crescents. Clinical details are shown in **Table 1**. One best section was placed in the center of a standard glass slide and stained with hematoxylin and eosin (H&E) and coverslipped, with standard protocol used in the routine practice of diagnostic pathology. De-identified slides labeled with serial numbers were sent for spatial transcriptomic profile data capture, at the University of Michigan Genomics Core Laboratory, Ann Arbor, Michigan. After uncoverslipping the slides, the tissue was de-crosslinked to release mRNA that was sequestered by formalin fixation. The standard protocol for probe hybridization on the Visium slides and data capture using 10x Genomics software is described in the **Supplementary Data File**. The study was approved by the Ohio State University Internal Review Board (IRB STUDY20251654).

**Table 1:** Kidney biopsy samples, Staphylococcus infection-associated glomerulonephritis (SAGN) (n=3); normal control kidney (NCK), n=1.

Patient	Age	Sex	Site of infection and (Pathogen)	Baseline S.cr.e	S.cr. at biopsy mg/dL	Blood on urinalysis	Serum C3, C4 (mg/dL)*	Glomeruli on LM (n)	% globally sclerotic	% crescents	Endocapillary hypercellularity	IF/TA	Diabetic glomerulosclerosis	IgA on IF	C3 on IF
SAGN Case 1	39	M	Endocarditis (MSSA)	0.9 (1 month before biopsy)	4	Large blood	Low C3*, normal C4	7	0	14%	present	Mild (10-20%	Absent	1+	2+
SAGN Case 2	60	F	Pneumonia (MRSA)	0.9 (1 month before biopsy)	8	Large blood	normal C3 and C4*	17	0	5%	present	Mild (20%)	Absent	1+	3+
SAGN Case 3	49	M	Pneumonia (MRSA)	0.6 (1 wk before biopsy)	6.5	20 RBCs/hpf	C3:24 C4:20	15	0	40%	Diffuse with intracapillary PMNs	Mild (10%)	Absent	1+	2+
NCK	45	M	N/A	N/A	N/A	N/A	N/A	54	0	0	absent	absent	Absent	N/A	N/A

MSSA, methicillin sensitive Staphylococcus aureus; MRSA, methicillin resistant Staphylococcus aureus; S.cr., serum creatinine; IF/TA, interstitial fibrosis and tubular atrophy (graded semiquantitatively 0, 1+ (<25%, 2+ (25 to 50%), 3+ (>50%); LM, light microscopy; IF, direct immunofluorescence staining, graded semi-quantitatively - 1+ mild, 2+ moderate, 3+ strong; hpf, high power field; LM, light microscopy. *Normal established reference ranges - Serum C3: 87-200 mg/dL; Serum C4: 18-52 mg/dL.

### Spatial transcriptomics data analysis with Seurat

Count matrices produced by spaceranger count were processed using the R package Seurat (v4.4) (13). Cells with low UMI count, very few expressed genes, or excessive mitochondrial gene load (indicative of dead/dying cells) were filtered out. scDblFinder was used to remove UMI doublets from the aggregate dataset (**Supplementary Figure 1B**) (14). Individual samples were then integrated using Seurat’s SCTransform-specific integration workflow. The cells were clustered by applying the K-nearest neighbors (KNN) graph based on PCA (principal component analysis) reduced space, followed by Louvain’s algorithm available in Seurat (v4.4). The cells were projected to a two-dimensional space using the Uniform Manifold Approximation and Projection (UMAP) dimensionality reduction technique. Annotation of computationally predicted clusters to biological cell type was performed manually using a combination of the following databases: CellMarker, CellMarker 2.0, panglaoDB, and czscience (15–19). Gene cluster markers of each population were detected by identifying significant differentially expressed genes between one population and the rest of the cells using the Wilcoxon test, available in the Seurat package (v4.4), including only genes expressed in at least 25% of the cells of either group. For differential gene expression analysis, we utilized both DESEQ2 and MAST algorithms.

### Annotation of cell populations in the kidney biopsies

The kidney biopsy sections were analyzed by transcriptomic and computational methods (**Supplementary Figure 1**). The quality of the Illumina sequencing data from the four kidney biopsy samples was assessed by total read count (the number of RNA molecules detected per cell; **Supplementary Figure 1**), feature count (number of genes detected per cell; **Supplementary Figure 1**), the mitochondrial content (percentage of mitochondrial genes, a marker for dead cells; **Supplementary Figure 1**), and the sequence reads from hemoglobin (as a proxy for blood contamination, **Supplementary Figure 1**). Data showed a low percentage of mitochondrial content and near-zero levels of hemoglobin across all samples, indicating good cell viability. To visualize the high-dimensional data, the UMAP method was applied, which resolved the cell populations into 12 distinct clusters with minimal overlap (**Supplementary Figure 1**). UMAP of the pooled reads from the four biopsies showed comparable distribution of cell populations (**Supplementary Figure 1**). Cell Ranger analysis followed by annotation tools in the Seurat R package allowed clustering and annotation of cell types using marker genes characteristic of each cell type shown as dot plots (**Supplementary Figure 1H**). The Human Protein Atlas and the databases CellMarker, CellMarker 2.0, panglaoDB, and czscience were used as reference for the marker genes (15–19). The resulting computational clusters assigned to biological cell types are shown in UMAP (**Supplementary Figure 1T**; UMAP).

### Cell communication network analysis using CellChat

To investigate cell communication patterns across the identified cell types and to achieve a better representation of the differences between control NCK and SAGN tissues, we used CellChat (20). CellChat is an R package designed to analyze intercellular communication networks from single-cell RNA sequencing (scRNA-seq) data through systematic identification and quantification of ligand–receptor interactions. CellChat utilizes a comprehensive database of known ligand–receptor pairs and their downstream signaling pathways to calculate the communication probabilities across sender and receiver clusters/cell types in the biopsy samples. We focused on the secreted signaling database and identified over-expressed genes (detailed procedure in **Supplementary Data**). Based on the gene expression, we calculated communication probabilities and aggregated them on a pathway level for downstream analysis. Each pathway is represented as a large vector of communication with aggregated probabilities for a given pathway across any sender cell type and any receiver cell type (e.g., Pathway_Sender_Receiver (**Table 1**). We used 8% as a cutoff for selection of significant communication probabilities following a ranked ordering of such probabilities. Based on the observed absolute differences in the calculated communication probabilities between SAGN and NCK biopsies, the communication nodes were graphed to highlight the order of putative importance (**Supplementary Figure 2**). Multiple dots of the same color represent repeated occurrence of the signaling [secreted phosphoprotein 1 (SPP1), etc.] among various cell clusters.

### Indirect immunofluorescence staining

This was performed on FFPE tissue sections with antibodies to SPP1/osteopontin, CK7, and CD10 (21, 22). Primary antibodies were purchased from Proteintech (SPP1 rabbit polyclonal), Sakura (CK7 mouse monoclonal, clone OV-TL-12/30), and Leica (CD10 mouse monoclonal, clone 56C6). Briefly, the staining protocol was as follows: Antigen retrieval was performed by using EDTA (pH 9) in a Biocare Decloaker. The primary antibody SPP1 was applied to sections at 1:100 for 1 h at room temperature after Serum Free protein block for 10 min. The secondary antibody Donkey anti-Rabbit 594 (red) at 1:100 was applied to slides for 30 min. Then, CK7 and CD10 primaries were applied to sections for 30 min at room temperature. The secondary antibody Alexa Fluor 488 (green) Goat anti-Mouse at 1:100 was applied for 30 min. Slides were coverslipped using Agilent Fluorescent mounting medium.

## Results

### Resident and immune cell clusters in the kidney biopsies

Following quality control steps, a total of ~2,000 cells from the healthy NCK biopsy and between 437 and 858 cells from each of the SAGN biopsies were identified. Their transcriptomes were retained for further analysis. Mapping of the reads to the H&E-stained sections of the kidney biopsies revealed uniform distribution of the cells. Spatial dim plots showing the organization of various cell types in NCK and SAGN kidneys together are shown in **Supplementary Figure 2**. The cells were classified into 12 major clusters, and transcriptomic identification of the cell types allowed a comparison of the cell composition in SAGN biopsies compared to NCK biopsy (**Supplementary Figure 2**). The highest variation between NCK and SAGN was observed in the immune cell and parietal epithelial cell (PEC) clusters (**Supplementary Figure 2**). Compared to NCK biopsy that showed 2.88% of immune cells, SAGN biopsy showed 5.9%–10% of immune cells. The proportion of immune cells relative to resident cells, however, was low (up to 10% of total cells), consistent with the microscopic observation that SAGN biopsies usually show only moderate patchy (not diffuse) interstitial inflammation. We simultaneously probed both resident kidney cell subsets and immune cells, but the latter were not further subdivided into individual subsets. In contrast, we observed diminished endothelial cells in the SAGN biopsies (2.5%–4.78%) compared to NCK control (9.35%). Proximal tubule stromal cells did feature in the transcriptomes of all four biopsy samples but without significant difference in abundance between NCK and SAGN kidneys. Although not a primary component of the nephron, this cell cluster consists of supporting mesenchymal stromal/progenitor cells, probably serving as an intermediate between tubular cells and the surrounding interstitium.

We constructed cell-to-cell communication networks among the 12 distinct cell clusters and ranked the networks based on the communication probabilities. This analysis revealed SPP1 as the predominant communication network among various cell types followed by vascular endothelial growth factor (VEGF), plasminogen (PLG), GRN, angiopoietin-like proteins (ANG PTL), and complement (**Supplementary Figure 2**).

### Complement activation in SAGN

**Figure 1:**
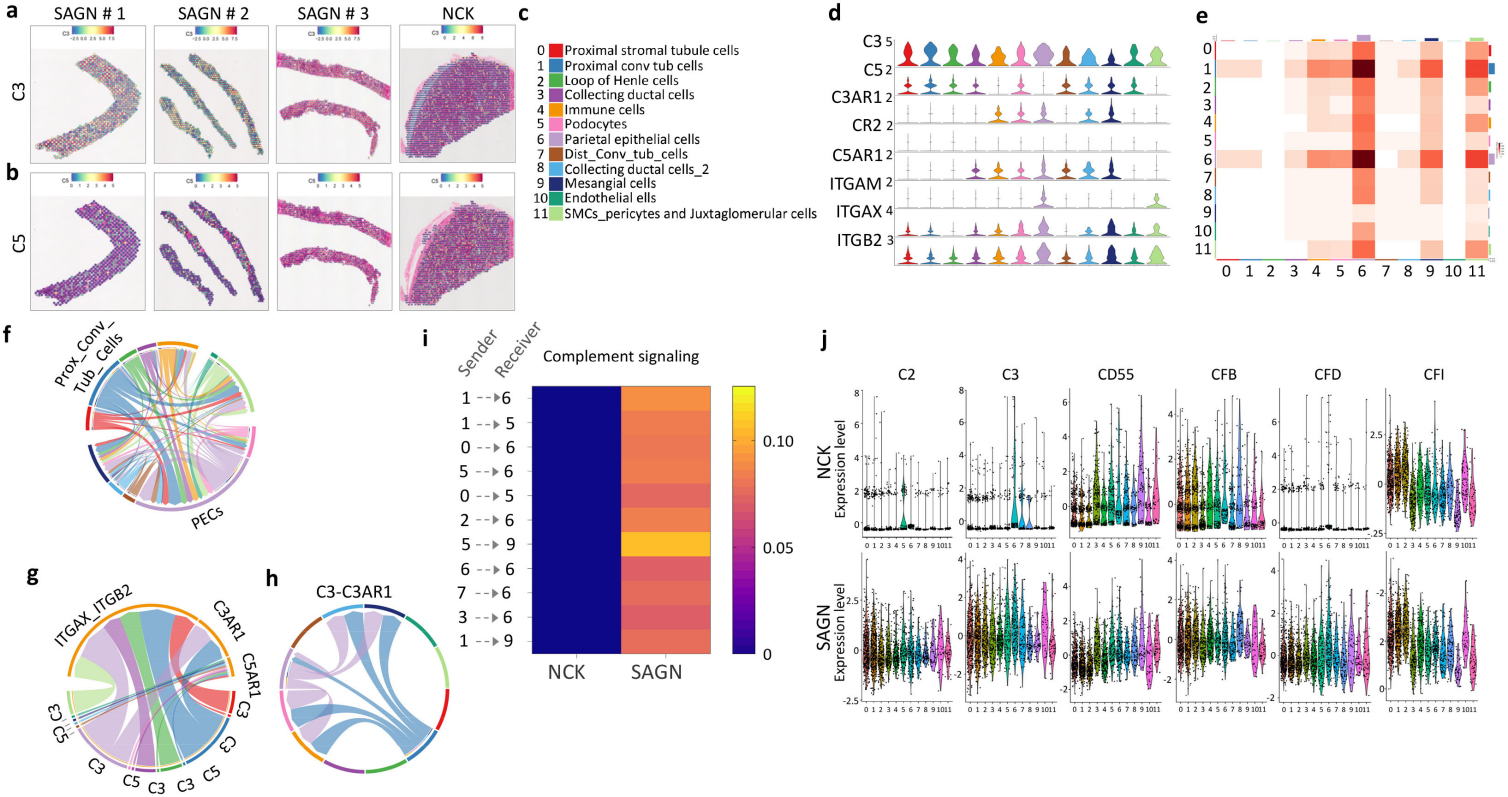
Spatial transcriptomic analysis identifies enriched complement network in SAGN. Expression of **(A)** C3 and **(B)** C5 complement markers is shown in discrete cellular compositions in SAGN and NCK biopsies. **(C)** The color code of cell types is shown. **(D)** Violin plot showing expression level of representative marker genes of complement broken down by cell clusters in combined NCK and SAGN biopsy samples. **(E)** Heatmap of complement communication network in SAGN samples shows PECs to be the major receivers of complement signal. PECs and proximal convoluted tubule cells are the major senders in the complement network. The *X*-axis indicates signal senders and the *Y*-axis shows signal receivers. **(F)** Chord diagram showing high levels of complement-mediated signaling between parietal epithelial cells, mesangial cells, proximal convoluted tubular cells, and pericyte smooth muscle cells, where the arc length is proportional to the strength of communication. **(G)** Chord diagram with complement-mediated signaling between C3/C5 and its cognate receptors ITGAX/ITGB2 and C3AR1/C5AR1 in immune cells. **(H)** Chord diagram showing communication probability between C3 and C3-associated receptor 1 (C3AR1) largely between proximal convoluted tubules, PECs, podocytes, immune cells, mesangial cells, and collecting duct cells. PECs act as senders and receivers. **(I)** Heatmap comparing top communication probabilities between NCK and SAGN biopsies. Communication probabilities are distinctly increased in diseased compared to normal kidneys. **(J)** Violin plots of ligand and receptor transcripts in each cell cluster in NCK (top panel) and SAGN kidney biopsies (bottom panel). All except CFI show an increased expression level in SAGN compared to NCK samples.

Mapping of complement components C3 (**Figure 1A**) and C5 (**Figure 1B**) on spatial dim plots showed an elevated expression in SAGN compared to NCK. Accordingly, our CellChat analysis revealed that the calculated communication probability for the complement network in the SAGN biopsy samples was markedly elevated compared to NCK biopsy (up to 9% compared to 0%, respectively) (**Figure 1I**; **Table 1**). Expression of C3, C5, and their receptors in NCK and SAGN is represented as violin plots (**Figure 1D**). Heatmap showing senders (*X*-axis) and receivers (*Y*-axis) of complement signal across individual cell clusters highlights PECs as the dominant signal receivers in SAGN specimens, with both paracrine and autocrine signaling loops (**Figure 1E**). Chord diagram depicts proximal convoluted tubule (PCT) and PEC clusters to be the major signaling axis followed by other cell clusters in a decreasing order (**Figure 1F**, represented as the arc length). The communication between C3, C5, and their receptors was visualized by the chord diagrams (**Figures 1G, H**). A heatmap constructed based on the ranked order of communication probabilities among various cell clusters between NCK and SAGN clearly illustrated the complement signaling enriched in SAGN, more commonly in PECs (cluster #6) either as senders or receivers, but undetectable in NCK (**Figure 1I**). Podocytes, mesangial cells and SMCs/pericytes, juxta-glomerular cells, and immune cells were receivers of complement signals in a decreasing order as well. Complement ligands—C2, C3, complement Factor D (CFD), complement Factor B (CFB), and CD55—showed significantly higher expression of mRNA transcripts in SAGN compared to normal kidneys (**Figure 1J**).

### SPP1–CD44 is the predominant signaling among resident and immune cells in SAGN

**Figure 2:**
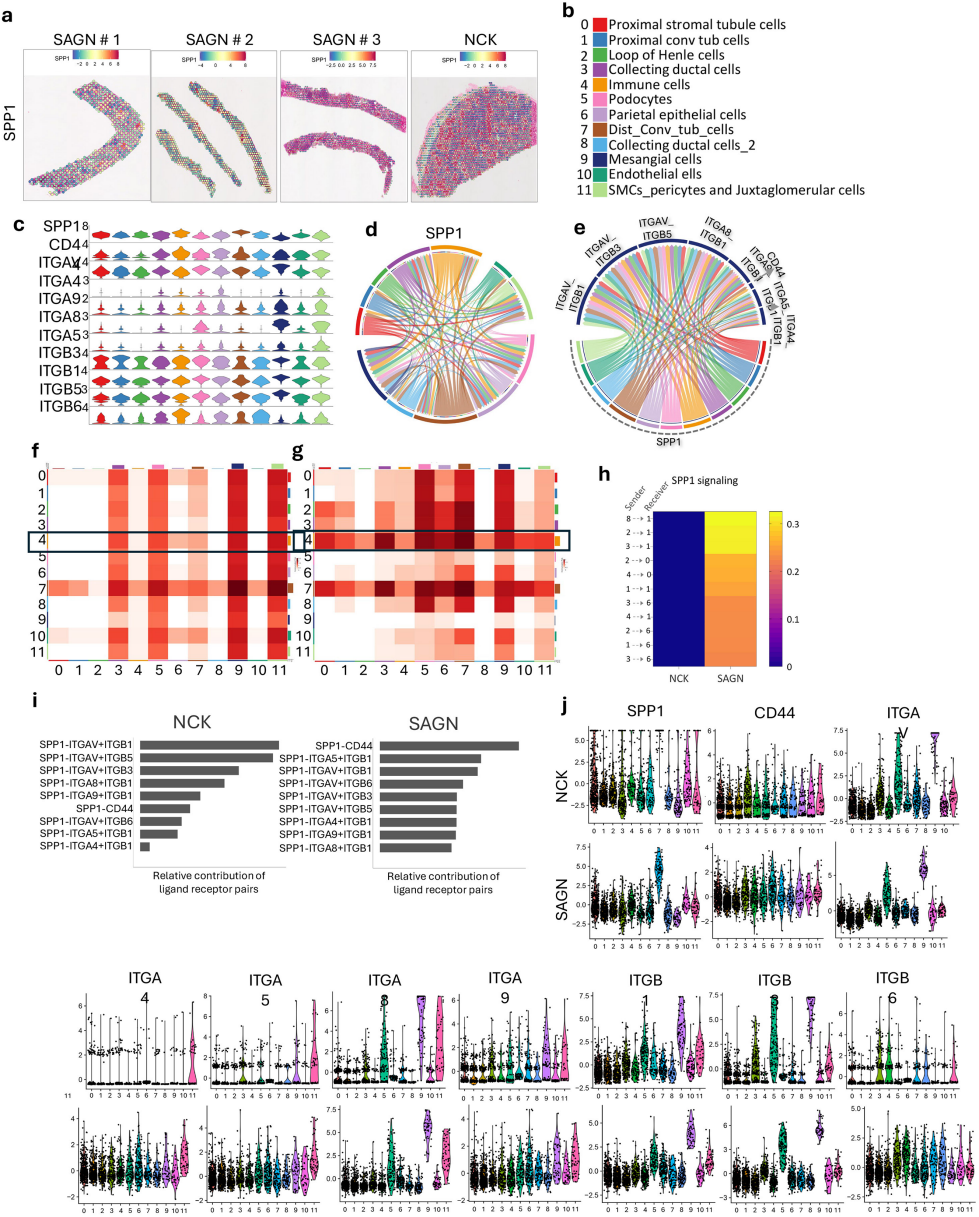
Secreted phosphoprotein 1 (SPP-1) network is the top enriched network in SAGN. **(A)** Expression of SPP-1 in discrete cellular compositions is shown in SAGN and NCK biopsies. **(B)** The color code of cell types is shown. **(C)** Violin plot showing overall expression level of representative marker genes of SPP-1 and its receptors broken down by cell clusters combining the NCK and SAGN biopsies together. **(D)** Chord diagrams showing the SPP-1-mediated signaling between various cell clusters combining all biopsy samples, where the arc length is proportional to the strength of communication. **(E)** Chord diagram shows mesangial cells as signal receivers with cognate receptors. **(F)** Heatmap of the SPP-1 communication in NCK showed the highest communication between mesangial (receivers) and distal convoluted tubular epithelial cells (senders), also involving smooth muscle cells, pericytes, and juxta-glomerular cells. Mesangial cells were the highest signal receivers in the normal kidney. The *X*-axis indicates signal senders and the *Y*-axis shows signal receivers. **(G)** Heatmap showing SPP1 communication in SAGN biopsies. There is markedly increased communication between various tubular segments, immune cells (as signal senders), and PECs and podocytes (as signal receivers). Autocrine signaling in distal convoluted tubule cells is also high. **(H)** Heatmap showing comparison of top communication probabilities between NCK and SAGN biopsies showing markedly increased SPP1 signaling to PECs in diseased kidneys. **(I)** Comparison of receptor usage and relative contribution of ligand–receptor pairs for SPP-1 signaling in NCK and SAGN biopsies is shown. Preferential signaling through CD44 is seen in diseased kidneys. **(J)** Violin plots showing transcript levels of SPP1 and SPP1 receptors in individual cell clusters, comparing NCK in the top panel and SAGN kidney biopsies in the bottom panel. Most receptor transcripts are increased in SAGN.

CellChat analysis showed that the SPP1 (also called osteopontin) network was the single most enriched network in our SAGN biopsies with the highest communication probability (~30%) and multiple ligand–receptor interactions (**Figures 2A–J**). Dim plots of H&E-stained sections of SAGN biopsies overlaid with the abundance of SPP1 transcripts showed constitutive expression in NCK and elevated expression in SAGN biopsies (**Figure 2A**). Expression of SPP1 and its constituent receptors across various cell types is shown as violin plots (**Figure 2C**). To deconvolute the SPP1 signaling into its constituent parts, we analyzed expression of SPP1, the multitude of its receptors, and their communication probabilities. Chord diagrams illustrating the interactions between the SPP1 ligand and its various receptors across various cell types are shown (**Figures 2D, E**). Heatmaps showing senders (*X*-axis) and receivers (*Y*-axis) of SPP1 signal across individual cell clusters in healthy control (NCK; **Figure 2F**) and SAGN biopsies (**Figure 2G**) were constructed based on communication probabilities. In NCK, SPP1 communication was found mainly between distal convoluted tubule cells (as senders, *Y*-axis), and mesangial cells, podocytes, and the cluster containing smooth muscle cells (SMCs), pericytes, and juxtaglomerular cells as signal receivers (**Figure 2F**; X-axis). A clear increase in communication probability was observed in immune cells in the SAGN compared to NCK biopsies (**Figures 2F, G** represented by a box). Other cell clusters also showing enriched SPP1 signaling compared to NCK were PCT, loop of Henle cells, and collecting ducts. They were strong senders, and PECs emerged as the strongest signal receivers. Podocytes also act as signal receivers. A heatmap constructed based on the ranked order of communication probabilities among various cell clusters between NCK and SAGN clearly illustrated that the SPP1 signaling enriched in SAGN, more commonly in PCT cells, loop of Henle cells, collecting ductal cells, and immune cells, whereas such interaction was undetected in NCK (**Figure 2H**). The relative contribution of each of the interactions between various SPP1 ligand–receptor pairs in NCK and SAGN identified a clear shift in ligand–receptor usage from SPP1-ITGAV+ITGB1 in NCK to SPP1–CD44 in SAGN (**Figure 2I**). Expression of SPP1 and its receptors in NCK and SAGN biopsies across various cell types showed a clear enrichment of transcripts CD44, ITGA4, ITGA5, and ITGB6 (**Figure 2J**). The complete list of calculated probabilities of SPP1 signaling is shown in **Table 1** (Sheet 2).

VEGF was the second most enriched network after SPP1 (**Supplementary Figure 3**). Transcripts of VEGF-A were found enriched in SAGN compared to NCK on spatial dim plots (**Supplementary Figure 3A**). **Supplementary Figure 3** shows overall distribution of VEGF ligands and receptor transcripts across all cell clusters in the four biopsy samples. Chord diagrams and heatmap depict mesangial cells and, to a lesser extent, podocytes as the major signal receivers, and they show not only paracrine but also autocrine cell signaling (**Supplementary Figure 3D**). In contrast to complement and SPP1, the ranked order of communication probability for VEGF signaling showed a decrease in the SAGN as compared to NCK kidney (**Supplementary Figure 3**). Increased VEGF-A transcripts were noted selectively in the mesangial cells of SAGN (**Supplementary Figure 3**). Constitutive expression was seen in the remaining cell clusters in both NCK and SAGN kidneys (**Supplementary Figure 3**). Placental growth factor (PGF), VEGF-C, and its cognate receptor Flt-4 as well as KDR (Flk-1) showed increased transcript levels in SAGN in most of the cell clusters but not specific to any single cell type (**Supplementary Figure 3**).

The remaining enriched pathways include Progranulin/Granulin, Plasminogen and PARs (proteinase-activated receptors), ANG PTL, and MIF (macrophage migration inhibitory factor) (**Supplementary Figure 2**). All these networks showed decreased communication probabilities in SAGN compared to NCK (data not shown).

### Differential gene expression analysis identified genes from enriched signaling networks

The differentially upregulated gene transcripts from these networks in SAGN versus NCK were complement factors C2, C3, Factor B, and Factor D; F2R/PAR1 (PLG receptor from the PARs network) across all cell groups; PGF, VEGFC, KDR, and FLT4 receptors (VEGF network); ANGPTL1 and ANGPTL4 ligands (ANGPTL network); and CXCR4 receptor (MIF network). The complete DGE tables are included in **Supplementary Table 2**.

### Immunofluorescence staining for SPP1 is increased in SAGN kidneys

**Figure 3:**
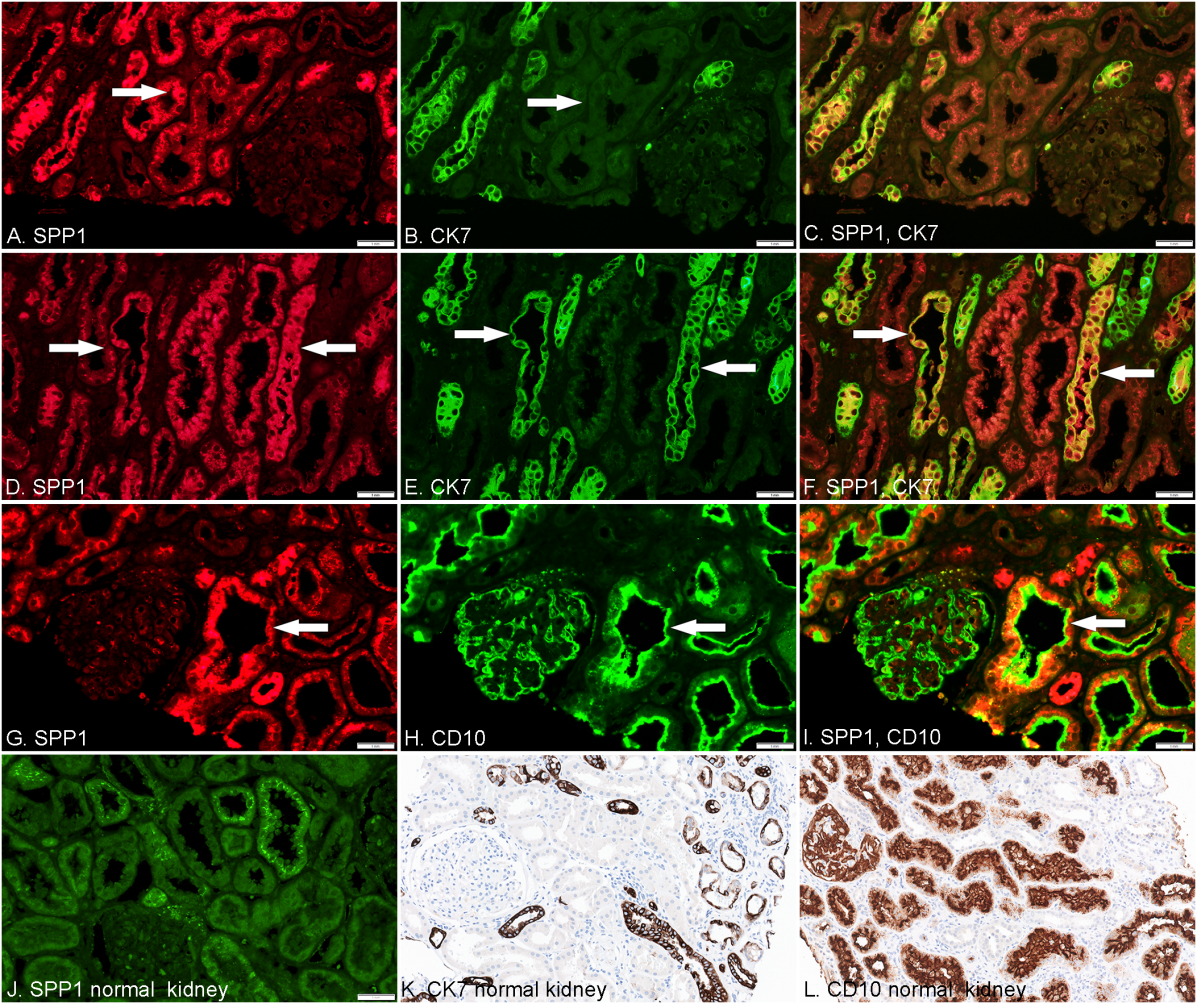
Validation of SPP1 expression by immunostaining. Indirect immunofluorescence (IF) staining for SPP1 in SAGN (red) and co-staining with CK7 and CD10 (markers for different tubular segments) in green. **(A)** SPP1 expression seen in proximal tubule segments (arrow). **(B)** Cytokeratin 7 (CK7) is absent in proximal tubules (arrow). **(C)** Lack of SPP1 and CK7 colocalization in proximal tubules. No glomerular expression for SPP1 seen. **(D)** SPP1 expression in distal and collecting tubules (arrow). **(E)** CK7 staining in distal and collecting tubules (arrow). **(F)** Double staining shows colocation in distal and collecting tubules (yellow). **(G)** SPP1 expression in proximal tubule segments but not in podocytes (arrow). **(H)** CD10 expression in glomerular podocytes and proximal tubules. **(I)** Double staining shows colocalization of SPP1 and CD10 in proximal tubule segments but not in podocytes. **(J)** Weak SPP1 staining (green) in normal kidney control in scattered distal tubules. **(K)** Immunohistochemical staining for CK7 in normal kidney control highlights distal and collecting tubule segments. **(L)** Immunohistochemical staining for CD10 in normal kidney control highlights podocytes in the glomeruli and proximal tubules (200×).

Staining for SPP1 and co-staining with CK7 (marker of distal tubules and collecting ducts) and CD10 (marker of proximal tubules)^21,22^, respectively, was performed. **Figures 3A–C** show the presence of SPP1, but the absence of CK7 in the proximal tubule segments and, therefore, the absence of co-staining (arrows). **Figures 3D–F** show the presence of SPP-1 and CK7 staining in the distal and collecting tubules demonstrating colocalization (arrows). **Figures 3G–I** highlight SPP1 staining in proximal tubules and colocalization with CD10 in the proximal tubules (arrow). CD10 stains podocytes as well, but SPP1 does not. This pattern confirms SPP1 expression in multiple tubular subsets, which were demonstrated as major signal senders in our transcriptomic data. Staining for SPP1 in NCK was observed only in a few distal tubule cross-sections and was much weaker in intensity (**Figure 3J–L**). Immunoperoxidase staining for CK7 and CD10 in normal kidney is shown in **Figures 3H, I**.

## Discussion

The goal of this study was to explore the spatial transcriptomic landscape in SAGN kidneys, which showed that even resident kidney cells (not only the professional immune cells) play an active role in the propagation of glomerular and tubular injury in SAGN. This is a pilot proof-of-concept study to assess the feasibility of CellChat with spatial transcriptomic technology on kidney biopsy tissue.

SAGN can show clinicopathologic heterogeneity. Biopsies can show a broad spectrum of histology ranging from mild mesangial hypercellularity, focal to diffuse endocapillary hypercellularity, with or without crescents. To minimize this variability and avoid skewing our spatial transcriptomic analysis toward outliers, we selected representative specimens using the following criteria:

(i) All the cases had culture-proven Staphylococcal infection; (ii) the infection was active and ongoing at the time GN developed; (iii) all three samples of SAGN kidneys included in this study showed diffuse endocapillary hypercellularity with focal crescents; and (iv) we did not separate out the crescentic and non-crescentic glomeruli within each biopsy. It is practically difficult to do and tends to reduce the number of cells per group available for analysis. Glomeruli that appear non-crescentic on the biopsy could, in fact, be harboring a segmental crescent not seen on a two-dimensional tissue section, and even the “non-crescentic” glomeruli may not be functionally normal, as these have endocapillary hypercellularity. (v) A vast majority of patients with SAGN are diabetic. We took care not to include biopsies with features of diabetic glomerulosclerosis, because we do not want transcriptomic signatures of the latter to confuse the picture.

We focused on the following: (i) annotation of computationally predicted cell clusters using a single-cell and spatial approach; (ii) identification of top enriched functional networks in normal and diseased kidney using gene expression and communication probabilities; and (iii) immunohistochemical confirmation for SPP1 protein expression in biopsy tissue. We attempted to identify *Staphylococcus aureus* transcripts as a test of direct infection of the kidney by using a custom hybrid genome reference consisting of concatenated human and *S. aureus* genomes (23). This did not reveal reads mapping uniquely to the *S. aureus*, consistent with the generally accepted view that SAGN/IRGN is not due to direct infection of the kidney, but a renal-limited immune-mediated complication triggered by infection elsewhere in the body. Methods are described in **Supplementary Data**.

Our findings indicate the active role of complement in SAGN and support earlier reports of *in vivo* complement synthesis in injured kidneys (24, 25). Upregulation of C3, CFD, and CFB transcripts (as opposed to C5) underscores the role of the proximal effector arm of the alternative complement pathway, similar to that seen in other complement-driven forms of GN such as IgA nephropathy, C3GN, and ANCA vasculitis showing better therapeutic efficacy with upstream complement inhibitors (targeting Factor B and C3) (26–28). PECs emerged as the major signal receivers in the complement (and SPP1) network, possibly providing an important link between PEC subsets and formation of glomerular crescents (29). Increased CFD expression by multiple resident cell types and immune cells in SAGN samples was also seen. The action of Factor D (cleavage of Factor B bound to [C3(H2O] or to C3b) is considered the rate-limiting step in the alternative pathway (AP) of complement activation. Factor D also participates in the amplification of the other complement activation pathways (30). Clinical trials, however, have not been very encouraging with CFD inhibitor Danicopan in IgA nephropathy or C3GN (31, 32). Complement receptor transcripts ITGAX and ITGB2 were also enriched in SAGN samples, but targeting them for therapeutic purposes is challenging. They may have a potential role as non-invasive diagnostic/prognostic markers as demonstrated in lupus nephritis and rheumatoid arthritis and as a susceptibility gene in IgA nephropathy (33–35).

The novel and major finding of our study is the enriched communication using the SPP1–CD44 axis in SAGN over and above the constitutional expression of SPP1 and SPP1-ITGAV+ITGB1 communication in NCK. Also, there is a shift of the major node of communication from the mesangial cells in NCK to PECs in SAGN kidneys. Extensive signaling from proximal, distal, and collecting tubule segments is seen in SAGN kidney (also confirmed by immunostaining; **Figure 3**).

SPP1 (or osteopontin) is a multi-functional phosphorylated glycoprotein, with a wide array of homeostatic cell functions including immune cell function, acting through a multitude of integrin cell surface receptors (36–38). Whether pathogenic or not may be context dependent (39, 40). The SPP1–CD44 axis seems to function as a conserved pro-inflammatory and pro-fibrotic signaling module across multiple tissues, most extensively characterized in the kidney, lung, and musculoskeletal system (41–45). In kidney injury, tubular epithelial cells upregulate CD44 and secrete SPP1, creating an SPP1-rich extracellular matrix that promotes chemotaxis and retention of macrophages and drives transition toward chronic interstitial inflammation. Tubule-derived exosomes containing N-terminal SPP1 activate fibroblast CD44, stimulating fibroblast proliferation, matrix production, linking Wnt/β-catenin activation to fibrosis progression in the transition from acute kidney injury to chronic kidney disease (40–43, 46). SPP1–CD44 did emerge as the most highly used ligand–receptor combination in SAGN kidneys (as compared to SPP1-ITGAV+ITGB1 in the normal kidney) in our study.

More recent transcriptomic studies on IgA nephritis, ANCA GN, lupus nephritis, and membranous nephropathy have also highlighted SPP1 expression (47–51). Okada et al. suggested the role of SPP1 in recruiting CD68-positive macrophages in human glomerulonephritis (52) and further demonstrated the effect of anti-sense SPP1 oligodeoxynucleotides and neutralizing SPP1 antibodies in suppressing interstitial monocyte infiltration and disease progression in animal models (53). SPP1 has recently been implicated in non-inflammatory kidney diseases as well, such as immune-tubular crosstalk and increased urinary excretion of receptor ITGB6 in patients with diabetic kidney disease (54–56). SPP1 thus shows a broad presence in kidney disease with potential as a urinary biomarker but may lack disease specificity. The hope of therapeutic targeting of SPP1 has emerged with recent results in pancreatic cancer literature implicating SPP1 (and receptor ITGB3) in increased cell plasticity (epithelial–mesenchymal) and tumor heterogeneity, promoting rapid cancer spread to distant sites (57, 58).

There were additional enriched networks, although communication probabilities were lower than those seen with SPP1 (**Supplementary Figure 2**). Validation of each of these is beyond the scope of this study, and their specific roles remain to be explored. One of them, however, was the VEGF network.

Disturbed VEGF signaling has been implicated in a major group of kidney disorders—thrombotic microangiopathy (TMA) (59–61)—but has never been explicitly linked to pathogenesis of GN. Decreased communication probability of the VEGF network in SAGN versus NCK, as demonstrated in our study, suggests loss of the protective effects of the VEGFA–VEGFR2 axis in this GN, suggesting abrogation of their endothelial cell protection during glomerulonephritis. We also noted a diminished number of endothelial cells in the SAGN kidneys compared to NCK (**Supplementary Figure 1**) (none of the biopsies showed the histological features of TMA). This may potentially contribute to or perpetuate capillary tuft injury in SAGN (59–61). The increased expression of PGF in SAGN is also interesting and could be a potential therapeutic target. PGF is known to selectively bind to VEGFR1 (Flt1) and to promote pro-inflammatory signaling, pathological angiogenesis, and the formation of heterodimers with VEGFA sequestering it from VEGFR2 (its cognate receptor), thereby abrogating the latter’s protective effects (59, 62, 63). VEGFC and FLT4 showed increased expression in SAGN, an axis thought to dampen inflammation by improving lymphatic drainage of inflammatory mediators from the site (64), suggesting a two-pronged effect of VEGF signaling in SAGN kidneys.

Decreased Progranulin/Granulin signaling has been previously shown to exert a reciprocal increase in pro-inflammatory signals (65) and, therefore, could potentially contribute to inflammation in SAGN along with SPP1 and complement. The role of the PLG/PPARs network remains to be explored, but the markedly increase in expression of F2R/PAR1 transcripts (a plasmin receptor) in SAGN kidneys may be an important finding as recently hypothesized in IRGN pathogenesis (1).

GN encompasses a group of disorders that cause glomerular inflammation and injury through an interplay of immune-mediated mechanisms, host characteristics, and environmental triggers, such as infections, drugs, malignancy, pregnancy, or autoimmunity. We have focused on SAGN and its top enriched cytokine/chemokine networks based on communication probabilities among resident kidney cell clusters and immune cells. Some common gene markers and the close physical proximity of cell clusters, particularly those within the glomerular tuft, can potentially lead to some degree of overlap.

Despite this pitfall, for the most part, single-cell transcriptomics greatly helped in furthering our understanding of cell–cell communication in SAGN. A combination of several transcriptomic networks probably culminates in the development and perpetuation of immunologic injury to the glomerular capillary tuft with important contributions from resident kidney cells in parallel with immune cells. Our study reinforced the role of complement in SAGN (particularly the proximal arm of AP). However, SPP-1 emerged as the dominant network with high SPP1–CD44 ligand–receptor usage. Additionally, VEGF signaling (or the lack of it) could be a player in the pathogenesis of GN, with some role for PLG/PARs. Commonalities in the usage of networks and ligand–receptor combinations in the immuno-pathogenesis of the seemingly heterogeneous subsets of glomerulonephritides could exist.

This is a pilot study with a small set of SAGN biopsies that were analyzed through a spatial transcriptomic platform. The main finding of SPP1 was confirmed by immunostaining in independent SAGN biopsies. However, our findings need to be validated in a larger set SAGN biopsies and control cases. This study is not powered to transition to a therapeutic intervention. Further studies elucidating these networks will be needed to derive specific targets for diagnosis and treatment.

## Data Availability

The datasets presented in this study can be found in online repositories. The names of the repository/repositories and accession number(s) can be found in the article/**Supplementary Material**.
