# Supplementary material for: Spatial transcriptomic analysis of kidney biopsies identifies activation of complement and SPP1 networks in Staphylococcus infection-associated glomerulonephritis
Source: Front Nephrol. 2026 Jul 17;6:1863912. doi: 10.3389/fneph.2026.1863912 (PMC13423683; doi:10.3389/fneph.2026.1863912)
Supplement: Supplementary file 1 [file DataSheet1.docx]

**Spatial transcriptomic analysis of kidney biopsies identifies** **SPP1-CD44 as the predominant signaling in Staphylococcus infection-associated glomerulonephritis (SAGN)**

Spyros Karaiskos^1#^, Luis Santana-Quintero^1#^, Cherri Bott^2^, Rahul Paul^1#^, Sergey V Brodsky^2^, Isabelle Ayoub^3^, Samir V Parikh^3^, Brad H Rovin^3^, Tibor Nadasdy^2^, Hira L Nakhasi^4#^, Sreenivas Gannavaram^4#*^, Anjali A Satoskar^2*^.

**Supplemental Data – Table of contents**

**Methods**

**Tissue Preparation for Visium CytAssist instrument**

Formalin-fixed paraffin embedded (FFPE) tissue blocks from four biopsy tissues samples (SAGN, n=3 and baseline zero-time living donor transplant biopsy representing normal control kidney, NCK, n=1) were sectioned at 5-micron thickness. All three samples of SAGN kidneys included in this study were cases with endocapillary hypercellularity with focal crescents. Clinical details are shown in Table 1 of main manuscript. One best section was placed in the center of a standard glass slide and stained with hematoxylin and eosin and coverslipped, with standard protocol used in the routine practice of diagnostic pathology. De-identified slides labelled with serial numbers were sent for spatial transcriptomic profile data capture, at the University of Michigan Genomics Core Laboratory, Ann Arbor, Michigan. After uncoverslipping the slides, the tissue was de-crosslinked to release mRNA that was sequestered by formalin fixation.

**Visium Cytassist Hybridization**

Human whole transcriptome probe panels, consisting of a pair of specific probes for each targeted gene, were added to the tissue. These probe pairs hybridize to their gene target and were then ligated to one another. The spatially barcoded, ligated probe products were released from the slide and PCR amplified. The probe products were further processed to generate an NGS-ready library. The Visium Spatial Gene Expression library was sequenced using standard short-read sequencers. Using license free software from 10X Genomics, data is processed and visualized using Space Ranger analysis pipelines and Loupe Browser visualization software.

**Data capture using 10x Genomics software**

During the Visium workflow, two main data types are captured: a tissue image and sequencing data for Visium FFPE with facilitated probe transfer using the Visium CytAssist instrument and third image is captured by the CytAssist to provide spatial orientation of the data. Two software tools provided by 10x Genomics, Space Ranger and Loupe Browser, were used to process and visualize these Visium data types. Space Ranger was used to process the input file types to align the Visium sequencing data with the image. Each Spatial Barcode with the associated UMIs (unique molecular identifiers) captured during the Visium workflow was assigned a spatial location in the tissue image. Space Ranger produced a variety of output files that were processed using R with the following packages: Seurat, spacexr.

**Spatial transcriptomics data analysis with Seurat**

Count matrices produced by spaceranger count were processed using the R package Seurat (v4.4).^8^ Cells with low UMI count, very few expressed genes or excessive mitochondrial gene load (indicative of dead/dying cells) were filtered out. scDblFinder was used to remove UMI doublets from the aggregate dataset (Fig. 1B-E).^S1^ Individual samples were then integrated using Seurat’s SCTransform specific integration workflow. The cells were clustered by applying the K-nearest neighbors (KNN) graph based on PCA (principal component analysis) reduced space, followed by Louvain’s algorithm available in Seurat (v4.4). The cells were projected to a two-dimensional space using the Uniform Manifold Approximation and Projection (UMAP) dimensionality reduction technique. Annotation of computationally predicted clusters to biological cell type was performed manually using a combination of the following databases: CellMarker, CellMarker 2.0, panglaoDB, czscience.^10,S2-S5^ Gene cluster markers of each population were detected by identifying significant differentially expressed genes between one population and the rest of the cells using Wilcoxon test, available in Seurat package (v4.4), including only genes expressed in at least 25% of the cells of either group. For differential gene expression analysis, we utilized both DESEQ2 and MAST algorithms.

**Annotation of cell populations in the kidney biopsies**

The kidney biopsy sections were analyzed by transcriptomic and computational methods (Supplementary Figure 1a). Quality of the illumina sequencing data from the four kidney biopsy samples was assessed by total read count (the number of RNA molecules detected per cell, Supplementary Figure 1b), feature count (number of genes detected per cell, Supplementary Figure 1c), the mitochondrial content (percentage of mitochondrial genes, a marker for dead cells, Supplementary Figure 1d) and the sequence reads from hemoglobin (as a proxy for blood contamination, Supplementary Figure 1e). Data showed a low percentage of mitochondrial content, near zero levels of hemoglobin across all samples indicating good cell viability. To visualize the high dimensional data, Uniform Manifold Approximation and Projection (UMAP) method was applied which resolved the cell populations into 12 distinct clusters with minimal overlap (Supplementary Figure 1f). UMAP of the pooled reads from the four biopsies showed comparable distribution of cell populations (Supplementary Figure 1g). Cell Ranger analysis followed by annotation tools in Seurat R package allowed clustering and annotation of cell types using marker genes characteristic of each cell type shown as dot plots (Supplementary Figure 1h-s). Human Protein Atlas and the following databases CellMarker, CellMarker 2.0, panglaoDB, czscience were used as reference for the marker genes. ^10,S2-S5^ The resulting computational clusters assigned to biological cell types are shown in UMAP (Supplementary Figure 1t).

**Cell Communication Network (CCN) analysis using CellChat**

To investigate cell communication patterns across the identified cell types and to achieve a better representation of the differences between control NCK and SAGN tissues, we used CellChat.^20^ CellChat utilizes a database of known ligand receptor pairs to calculate the communication probabilities across sender and receiver clusters/cell types in the biopsy samples. We focused on the secreted signaling database and identified over-expressed genes (detailed procedure in Supplemental data). Based on the gene expression we calculated communication probabilities and aggregated them on a pathway level for downstream analysis. Each pathway is represented as a large vector of communication with aggregated probabilities for a given pathway across any sender cell type and any receiver cell type (e.g. Pathway_Sender_Receiver (Supplemental table 1). We used 8% as a cut-off for selection of significant communication probabilities following a ranked ordering of such probabilities. Based on the observed absolute differences in the calculated communication probabilities between SAGN and NCK biopsies, the communication nodes were graphed to highlight the order of putative importance (Fig. 1c). Multiple dots of the same color represent repeated occurrence of the signaling (SPP1 etc.,) among various cell clusters.

**Curation of custom reference and Read processing for *Staphylococcus aureus***

For the alignment step, genomic sequences and .gtf files for *Homo Sapiens* (assembly GRCh38) were merged with *Staphylococcus aureus* (version GCA_000009645.1) to create a custom reference. The annotation information for *S. aureus* microbe was appended to the end of the human annotation file. The merged genome and annotation custom reference was then processed by spaceranger/cellranger mkgtf and then spaceranger/cellranger mkref (version 2.1.0), using the default parameters, to create the appropriate genome index files. All libraries were aligned to the custom curated reference.

Base calling was performed using spaceranger mkfastq with default parameters. The sequenced reads for each sample were processed separately using spaceranger count against the host/microbe merged genome and the corresponding merged annotation gtf file. Formal resolution of this issue requires targeted enrichment of microbial transcripts which is out of the scope of this study since the barcodes supplied by 10X Genomics are not designed to enrich microbial transcripts.

**Cell Chat communication Software**

CellChat is an R package designed to analyze intercellular communication networks from single-cell RNA sequencing (scRNA-seq) data through systematic identification and quantification of ligand-receptor interactions. The software employs a comprehensive database of known ligand-receptor pairs and their downstream signaling pathways, using probabilistic models to determine the likelihood and strength of communication between cell populations while accounting for inherent noise in single-cell data. CellChat generates quantitative metrics of communication strength and creates network visualizations to map intercellular signaling patterns, enabling identification of disrupted communication pathways between experimental conditions and facilitating discovery of potential therapeutic targets through comparative analysis of cell-cell communication networks. Here we used the software to extensively examine NCK and SAGN biopsies. We identified all of the communication nodes across all biopsies, and we present the most variable networks with respect to NCK vs SAGN biopsies.

To achieve compatibility, we treated the spatial RNAseq matrices as single cell RNAseq matrices and loaded the data using Read10X function instead of Load10X_Spatial. Computational clusters were confirmed to match the exact clusters produced when using the Load10X_Spatial results function. We focused on secreted signaling communication database composed of thousands of ligand-receptor combinations tailored for single cell RNA-seq datasets. We calculated the communication probability for each ligand receptor pair across all samples and harmonized the dataset by imputing 0 communication probability for instances where a pair of ligand-receptor communication was not detected. The communication probability for each ligand-receptor interaction was averaged for each distinct network/pathway observed in this study.

**Indirect immunofluorescence staining**

This was performed on FFPE tissue sections with antibodies to SPP1/osteopontin, CK7, CD10.^12,S6^ Primary antibodies were purchased from Proteintech (SPP1 rabbit polyclonal), Sakura (CK7 mouse monoclonal, clone OV-TL-12/30), and Leica (CD10 mouse monoclonal, clone 56C6). Briefly the staining protocol was as follows: Antigen retrieval was performed by using EDTA pH 9 in a Biocare Decloaker. Primary antibody, SPP1 was applied to sections at 1:100 for 1 hour at room temp after Serum Free protein block 10 minutes. Secondary antibody, Donkey anti Rabbit 594 (red) at 1:100 was applied to slides for 30 minutes. Then CK7 and CD10 primaries were applied to sections for 30 minutes at room temp. Secondary antibody, Alexa Fluor 488 (green) Goat anti Mouse at 1:100 was applied for 30 minutes. Slides were coverslipped using Agilent Fluorescent mounting medium.

**Supplementary Results**

Following quality control steps, a total of ~2000 cells from the healthy NCK biopsy and between 437 and 858 cells from each of the SAGN biopsies were identified. Their transcriptomes were retained for further analysis. Mapping of the reads to the H&E-stained sections of the kidney biopsies revealed uniform distribution of the cells. Spatial dim plots showing the organization of various cell types in normal control kidney (NCK) and SAGN kidneys together are shown in Fig.1A. The cells classified into 12 major clusters, and transcriptomic identification of the cell types allowed a comparison of the cell composition in SAGN biopsies compared to NCK biopsy (Fig 1b). The highest variation between NCK and SAGN was observed in the immune cell and parietal epithelial cell (PECs) clusters (Fig.1b). Compared to NCK biopsy that showed 2.88% of immune cells, SAGN biopsy showed 5.9-10% of immune cells. The proportion of immune cells relative to resident cells, however, was low (up to 10% of total cells), consistent with the microscopic observation that SAGN biopsies usually show only moderate patchy (not diffuse) interstitial inflammation. We simultaneously probed both resident kidney cell subsets and immune cells, but the latter were not further subdivided into individual subsets. In contrast, we observed diminished endothelial cells in the SAGN biopsies (2.5-4.78%) compared to NCK control (9.35%). Proximal tubule stromal cells did feature in the transcriptomes of all four biopsy samples but without significant difference in abundance between NCK and SAGN kidneys. Although not a primary component of the nephron, this cell cluster consists of supporting mesenchymal stromal/progenitor cells, probably serving as an intermediate between tubular cells and the surrounding interstitium.

**Complement and complement receptors**

CFD transcripts were expressed by multiple resident cell types and immune cells in SAGN samples. The action of Factor D (cleavage of Factor B bound to [C3(H2O] or to C3b) is considered the rate limiting step in the alternative pathway (AP) of complement activation and the C3 amplification loop. Factor D also participates in the amplification of the other complement activation pathways - the classical pathway (CP) and lectin pathway (LP).^S25,S26^ In the systemic circulation, pro-Factor D (synthesized and secreted mainly from adipocytes as adipsin) needs to be cleaved to form the mature active form of Factor D under the control of MASP3, a player in the LP.^S26^ It is not known whether this applies to locally synthesized CFD within the kidney. Clinical trials, however, have not been very encouraging with CFD inhibitor Danicopan in IgA nephropathy or C3GN.^S27^

Complement receptor transcripts ITGAX and ITGB2 were found to be enriched in SAGN samples. These have shown potential as non-invasive markers to monitor disease activity in lupus nephritis.^S9^ *ITGAM-ITGAX* locus on 16p11.2 (with specific polymorphisms) is reported to be one of the susceptibility genes in IgA nephropathy in both European and Chinese patient cohorts.^S10^ Due to the diverse roles of these complement receptors in immune cell trafficking and scavenger function for denatured proteins and nucleic acids, targeting them for therapeutic purpose can be challenging. But they may have a potential role as diagnostic markers in autoimmune disease. ITGAX has been shown to be a treatment biomarker to predict response to TNF inhibitor drugs in rheumatoid arthritis.^S11^

**Diminished VEGF signaling detected in SAGN**

VEGF was the second most enriched network after SPP1 (Supplementary Figure 1c). Transcripts of VEGF-A were found enriched in SAGN compared to NCK on spatial Dim plots (Supplementary Figure 3a, b). Fig. 3C shows overall distribution of VEGF ligands and receptor transcripts across all cell clusters in the four biopsy samples. Chord diagrams and heatmap depict mesangial cells, and to a lesser extent podocytes, as the major signal receivers and they not only show paracrine but also autocrine cell signaling (Supplementary Figure 3 d, e, f). In contrast to complement and SPP1, ranked order of communication probability for the VEGF signaling showed a decrease in the SAGN as compared to NCK kidney (Supplementary Figure 3 g). Increased VEGF-A transcripts were noted selectively in the mesangial cells of SAGN (Supplementary Figure 3 h). Constitutive expression was seen in the remaining cell clusters in both NCK and SAGN kidneys (Supplementary Figure 3h). Placental growth factor (PGF), VEGF-C and its cognate receptor Flt-4 as well as KDR (Flk-1) showed increased transcript levels in SAGN in most of the cell clusters but not specific to any single cell type (Supplementary Figure 3h).

**Differential gene expression** (**DESeq2) analysis identified genes from enriched signaling networks.**

The differentially upregulated gene transcripts from these networks in SAGN versus NCK were – complement factors C2, C3, Factor B and Factor D; F2R/PAR1 (PLG receptor from the PARs network) across all cell groups, placental growth factor (PGF), VEGFC, KDR and FLT4 receptors (VEGF network), ANGPTL1, ANGPTL4 ligands (ANGPTL network), and CXCR4 receptor (MIF network). The complete DGE tables are included in Supplementary Table 2.

**Supplementary Figure Legends**

**Supplementary Figure 1)** Spatial transcriptomic analysis reveals the distribution of cell types in normal control kidney (NCK) and Staphylococcus-associated glomerulonephritis (SAGN) biopsies. a) Experimental design - residual FFPE tissues from diagnostic SAGN biopsies (n=3) and a control pre-implantation kidney transplant biopsy from a living donor (n=1) were used to perform scRNA-Seq using 10x Genomics. b) nCount or total number of transcripts with unique molecular identifiers from illumina sequencing, c) nFeature or total number of genes, d) % mitochondrial transcripts and E) % of hemoglobin transcripts are shown. f) UMAP plot from scRNA-Seq using Seurat package in R studio and g) distribution of cells from NCK and SAGN biopsies into 12 clusters by unsupervised clustering are shown. h-s) Dot plots depicting the expression of the top 50 marker genes used to characterize the 12 cell clusters are shown. t) Annotation of cell types based on the expression of marker genes is shown. It correlates with the distribution of cell clusters from NCK and SAGN biopsies by unsupervised clustering and the scRNA-Seq UMAP plot.

We did not separate out the crescentic and non-crescentic glomeruli within each biopsy, for several reasons: i) it is practically difficult to do and tends to reduce the number of cells per group available for analysis ii) glomeruli that appear non-crescentic on the biopsy, could in fact be harboring a segmental crescents not seen on a two-dimensional tissue section; and iii) Even the “non-crescentic” glomeruli may not be functionally normal, as these have endocapillary hypercellularity.

Supplementary Figure 2). Spatial dim plots showing the organization of various cell types in NCK and SAGN kidney biopsies. a) Discrete cellular compositions identified based on marker gene expression are indicated in SAGN and NCK kidney biopsies. b) The number of cells in each cluster and in each biopsy sample, is catalogued, including percent of total. c) Communication nodes based on the observed absolute differences in the calculated communication probabilities between SAGN and NCK biopsies are in the order of putative importance. Select communications are highlighted in color.

Supplementary Figure 3). VEGF network shows reduced communication in SAGN versus normal kidney. a. VEGF -A expression in discrete cellular compositions in SAGN and NCK biopsies. b. The color code of cell types is shown. c. Violin plot showing expression level of representative marker genes of the VEGF network broken down by cell clusters in combined NCK and SAGN biopsy samples. d. Chord diagram showing VEGF-mediated signaling between various cell clusters where the arc length is proportional to the strength of communication. e. Chord diagram showing array of ligands and cognate receptors distributed on mesangial cell cluster, contributing to autocrine signaling loops and paracrine signaling from immune cell cluster. f. Heat map of VEGF communication network shows high communication probability through mesangial cells and podocytes. Autocrine loops are also seen within these cell clusters g. Heat map showing comparison of top communication probabilities between NCK and SAGN biopsies for the VEGF network, showing markedly decreased signals in the diseased kidneys. h. Violin plots of VEGF-A, VEGF-C, PGF, FLT1, FLT4 and KDR receptor expression broken down by cell clusters in NCK and SAGN biopsies. VEGF-C, PGF, KDR and FLT4 did show upregulation in SAGN/diseased kidneys.

**Supplementary Table 1** Cell Communication probabilities (Excel spread sheet uploaded separately).

**Supplementary Table 2** Differential gene expression (Excel spread sheet uploaded separately).
