# Supplementary figures and images for: Spatial transcriptomic analysis of kidney biopsies identifies activation of complement and SPP1 networks in Staphylococcus infection-associated glomerulonephritis
Source: Front Nephrol. 2026 Jul 17;6:1863912. doi: 10.3389/fneph.2026.1863912 (PMC13423683; doi:10.3389/fneph.2026.1863912)

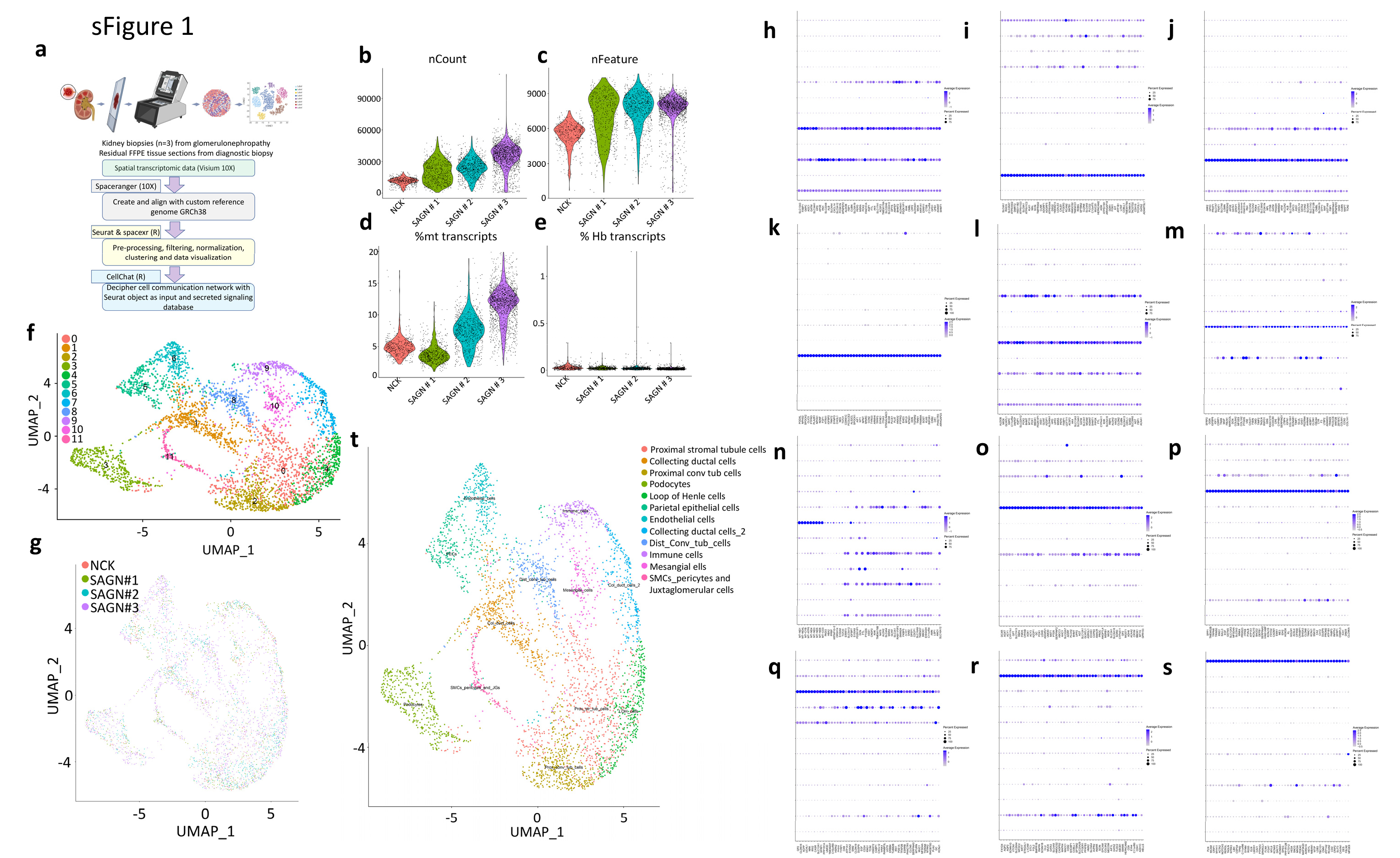

Supplement: Supplementary file 2 [file Image1.tif]

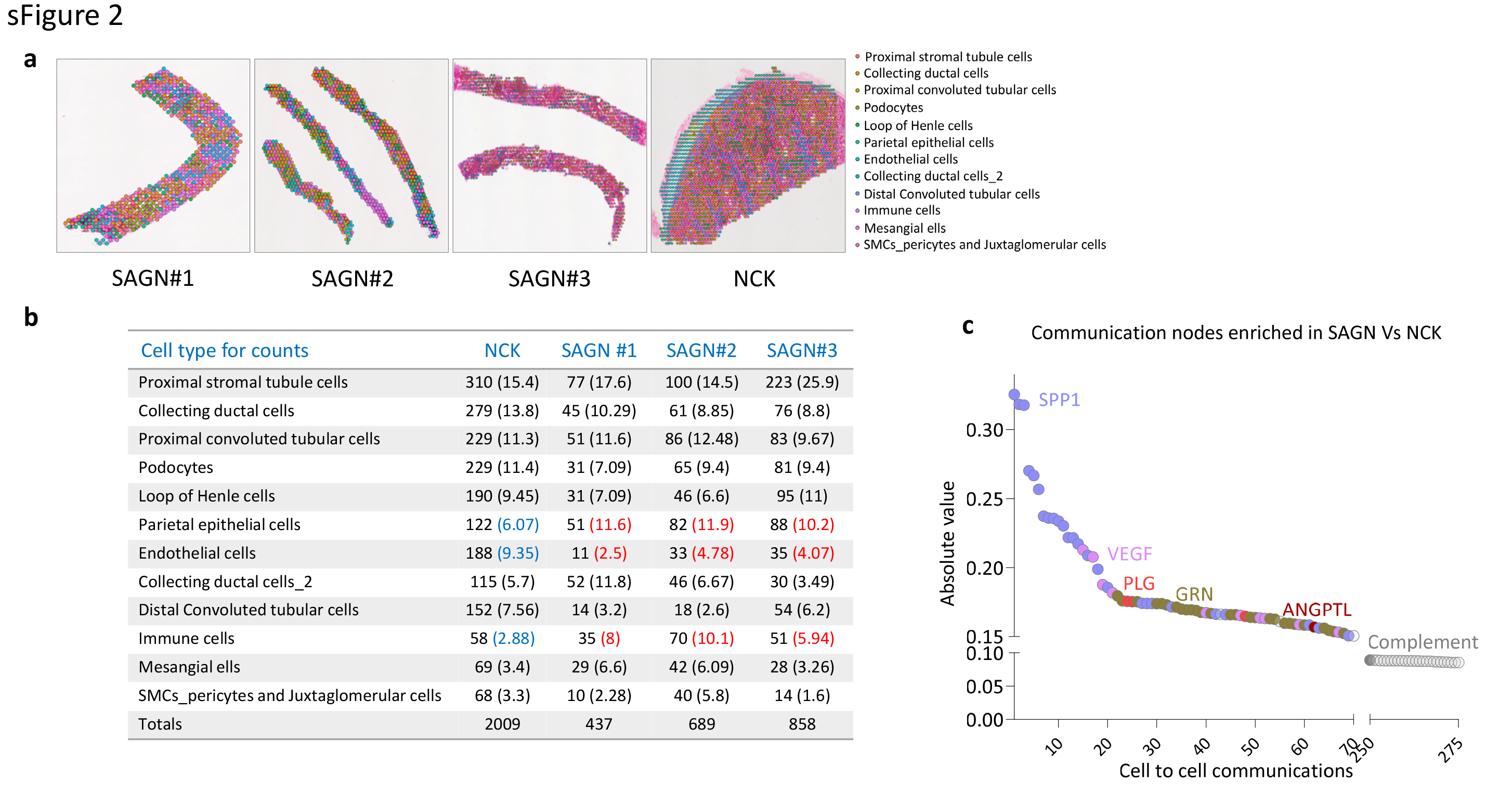

Supplement: Supplementary file 3 [file Image2.tif]

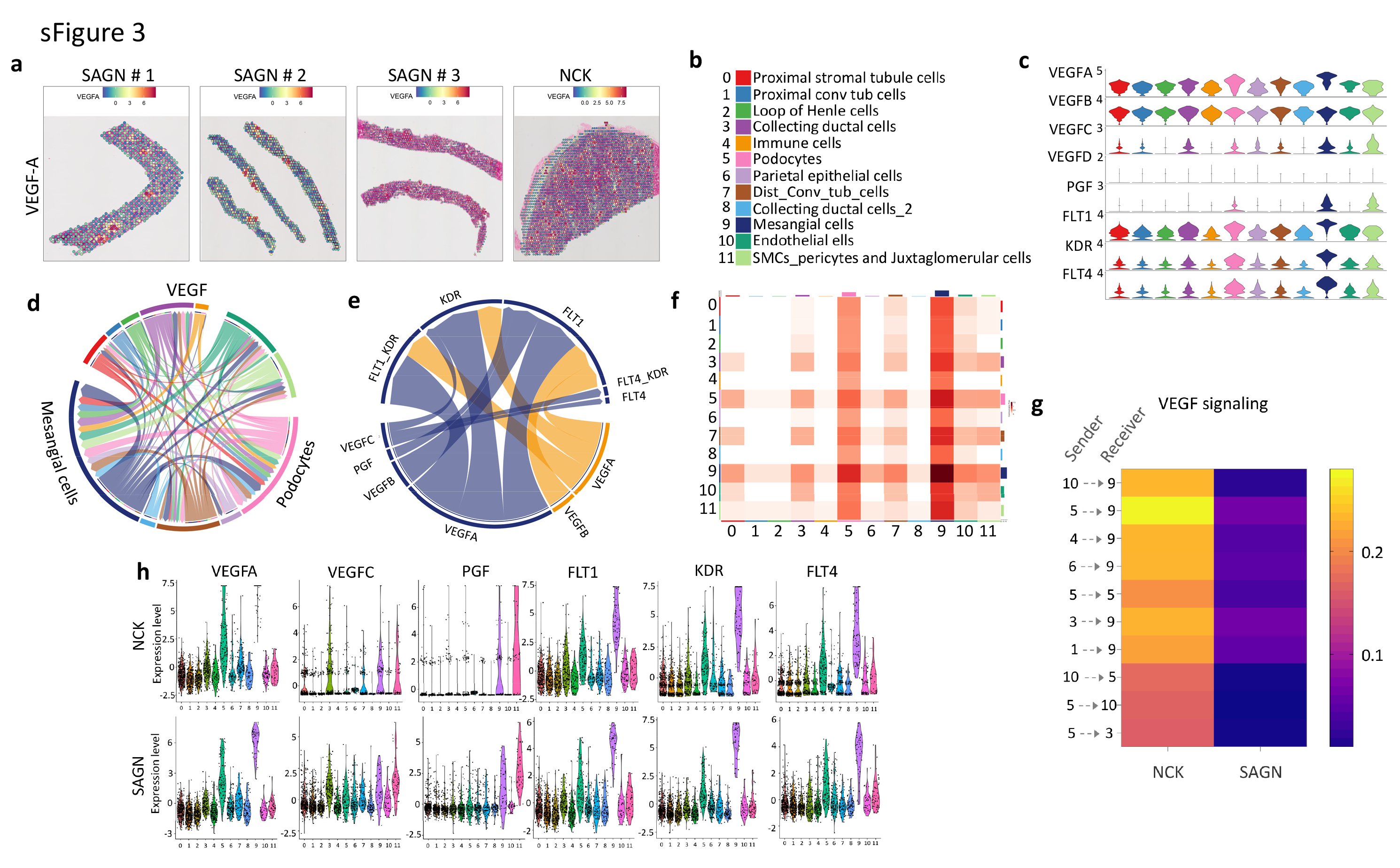

Supplement: Supplementary file 4 [file Image3.tif]
